# Fortschritte der Teilhabeforschung. Eine wissenschaftssoziologische Betrachtung ihrer Institutionalisierung

**DOI:** 10.1007/s00103-026-04275-x

**Published:** 2026-07-07

**Authors:** Markus Schäfers, Gudrun Wansing

**Affiliations:** 1 Fachbereich Sozialwesen, Hochschule Fulda, Leipziger Straße 123, 36037 Fulda, Deutschland; 2 Institut für Rehabilitationswissenschaften, Humboldt-Universität zu Berlin, Unter den Linden 6, 10099 Berlin, Deutschland

**Keywords:** Behinderung, Teilhabeforschung, Wissenschaftssoziologie, Institutionalisierung, Transdisziplinarität, Disability, Participation research, Sociology of science, Institutionalization, Transdisciplinarity

## Abstract

Teilhabeforschung hat sich als ein interdisziplinäres Forschungsfeld herausgebildet, das sich mit der gesellschaftlichen Teilhabe von Menschen mit Behinderungen befasst. Mehr als ein Jahrzehnt nach der Gründungs- und Aufbruchsphase dieses Forschungsfeldes stellt sich die Frage nach dem aktuellen Stand seiner Entwicklung. Diese narrative Übersicht analysiert die Fortschritte der Teilhabeforschung aus wissenschaftssoziologischer Perspektive und rekonstruiert sie als Prozess fortschreitender Institutionalisierung. Die Analyse struktureller, sozialer, epistemischer und politisch-gesellschaftlicher Aspekte zeichnet ein ambivalentes Bild. Insbesondere die Netzwerkbildung und die Herausbildung einer transdisziplinären epistemischen Kultur sprechen für deutliche Institutionalisierungsfortschritte. Zudem besitzt das Forschungsfeld eine hohe politische Relevanz. Die strukturelle Verankerung der Teilhabeforschung im Wissenschaftssystem ist bislang jedoch nur gering ausgeprägt.

## Abstract (en)

Participation research has emerged as an interdisciplinary field of research that focuses on the social participation of people with disabilities. More than a decade after its founding and initial phase, the question arises as to the current state of its development. This article examines the progress of participation research from a sociological perspective and reconstructs it as a process of progressive institutionalization. The analysis of structural, social, epistemic, and sociopolitical aspects paints an ambivalent picture. In particular, the network formation and the emergence of a transdisciplinary epistemic culture point to significant progress in institutionalization. Furthermore, the field of research has high political relevance. However, the structural anchoring of participation research within the academic system has so far been only modest.

## Einleitung

Der Begriff der Teilhabe hat sich in den vergangenen zwei Jahrzehnten sowohl in der deutschen Behindertenpolitik als auch in wissenschaftlichen Disziplinen, die sich mit Behinderung beschäftigen, zu einem zentralen Orientierungspunkt entwickelt [1, 2]. Seine zunehmende Bedeutung ist wesentlich auf das Sozialgesetzbuch IX, die Internationale Klassifikation der Funktionsfähigkeit, Behinderung und Gesundheit (ICF) der Weltgesundheitsorganisation [3] sowie die UN-Behindertenrechtskonvention (UN-BRK) zurückzuführen. Der Teilhabebegriff markiert einen paradigmatischen Wandel: weg von defizitorientierten, individualistischen und medizinisch dominierten Perspektiven auf Behinderung, hin zu einem sozialkontextuellen Verständnis, das die Wechselwirkung zwischen individuellen Voraussetzungen und gesellschaftlichen Barrieren in den Mittelpunkt rückt [4]. Die Etablierung des Teilhabebegriffs verlangt nach einer Neuorientierung des Wissenschaftsbetriebs und hat zur Entstehung von Teilhabeforschung als eigenem Forschungsfeld geführt, das sich „im Werden“ [5, S. 1] befindet. Es untersucht die gesellschaftlichen Teilhabebedingungen und Möglichkeitsräume der Lebensführung von Menschen mit Behinderungen sowie die Verwirklichung ihrer Teilhabe. Der Beitrag betrachtet die Entwicklung von Teilhabeforschung aus einer wissenschaftssoziologischen Perspektive. Im Mittelpunkt steht die Frage, welche Fortschritte ihrer Institutionalisierung im deutschsprachigen Raum zu erkennen sind. Dafür werden vier Dimensionen herangezogen: strukturelle Verankerung, soziale Vernetzung, epistemische Kultur und politisch-gesellschaftliche Problemrelevanz.

## Institutionalisierung der Teilhabeforschung

Als Institutionalisierung soll der Prozess der „Verstetigung, Sichtbarmachung und Absicherung (…) als Lehr- und Forschungsgebiet im Wissenschaftssystem“ [6, S. 895] verstanden werden. Interdisziplinäre Forschungsfelder können sich auf unterschiedlichen Wegen institutionalisieren. In der Wissenschaftsforschung werden dazu verschiedene Kriterien beleuchtet (vgl. exemplarisch für die berufliche Rehabilitationsforschung [7]; für die Disability Studies [8]; für die Frauen- und Geschlechterforschung [6, 9, 10]). Es lassen sich folgende Dimensionen unterscheiden: 1. *strukturelle* (z. B. Professuren, Forschungseinrichtungen, finanzielle Ressourcen), 2. *soziale* (z. B. Kommunikation, Kooperation und Vernetzung) und 3. *epistemische* (z. B. Erkenntnisfragen, Begriffsverständnis, methodologische Orientierungen). Dabei ist von einem Zusammenhang zwischen institutionellen Bedingungen und der Ausrichtung der Wissensproduktion auszugehen [11, 12]. Darüber hinaus ist 4. die *Relevanz* entscheidend, die dem Forschungsproblem politisch-gesellschaftlich zugesprochen wird, weil sie reguliert, welche Ressourcen zur Problembearbeitung zur Verfügung gestellt werden [13].

Im Folgenden werden diese vier Dimensionen auf die Teilhabeforschung bezogen, um ihren Institutionalisierungsgrad einzuschätzen.

### Strukturelle Dimension

Die strukturelle Dimension der Institutionalisierung zeigt, inwiefern Teilhabeforschung im etablierten Wissenschaftssystem verankert ist und dort strukturbildend wirkt. Als Indikatoren gelten z. B. eingerichtete Professuren, Forschungsstellen, Programme der Forschungsförderung und Forschungsinfrastruktur.^1^

In Deutschland ist derzeit keine dauerhaft eingerichtete Professur mit *Teilhabeforschung* als explizitem Schwerpunkt in der Denomination sichtbar. In der jüngeren Vergangenheit gab es an der Hochschule Ravensburg-Weingarten eine Professur *Angewandte Sozialarbeitswissenschaft, Schwerpunkt Teilhabeforschung* (2018–2021), an der Evangelischen Hochschule für Soziale Arbeit & Diakonie in Hamburg eine Professur *Disability Studies und Teilhabeforschung* (2020–2025). Beide Professuren waren innerhalb der Sozialen Arbeit angesiedelt und wurden zwischenzeitlich inhaltlich umgewidmet bzw. nicht erneut berufen. Im Rahmen des Fördernetzwerks Interdisziplinäre Sozialpolitikforschung (FIS) ist derzeit eine Stiftungsprofessur *Sozialpsychiatrische Teilhabeforschung* an der Universität Leipzig (Klinik für Psychiatrie) eingerichtet, die vom Bundesministerium für Arbeit und Soziales (BMAS) befristet finanziert wird [14].

Der Begriff *Teilhabe* hingegen lässt sich bei einer Reihe von Professuren im Kontext von Behinderung als Bestandteil der Denomination feststellen, vor allem mit einer disziplinären Verortung in den Rehabilitations- und Gesundheitswissenschaften, der Sozialen Arbeit und Pädagogik, auch in der Rechtswissenschaft: z. B. *Rehabilitation und Teilhabe* (Hochschule Merseburg), *Behinderung, Inklusion und Soziale Teilhabe* (Universität Kassel), *Medizinische Versorgung von Menschen mit Behinderung und Teilhabebeschränkungen* (Universität Augsburg). Darüber hinaus gibt es zahlreiche weitere Professuren, die thematisch stark an Teilhabeforschung anschließen dürften, auch wenn ihre Denominationen andere Begriffe verwenden, wie in den Erziehungswissenschaften z. B. Inklusion.

Auch hochschulische Forschungsstellen sind ein Gradmesser der Institutionalisierung von Forschungsfeldern. Eine explizite Schwerpunktsetzung auf Teilhabeforschung weisen der *Forschungsverbund für Sozialrecht und Sozialpolitik (FoSS)* der Universität Kassel und der Hochschule Fulda mit der *Arbeitsgruppe Teilhabeforschung* (seit 2013; [15]), das *Institut Mensch, Technik und Teilhabe *der Hochschule Furtwangen (seit 2015) sowie das *Institut für Teilhabeforschung* der Katholischen Hochschule Nordrhein-Westfalen (seit 2016) auf [16]. Das *Zentrum für Disability Studies und Teilhabeforschung (ZeDiSplus*; seit 2005) der Universität Hamburg bzw. Evangelischen Hochschule des Rauhen Hauses ist den Disability Studies zuzurechnen, führt jedoch auch Teilhabeforschung im Namen. Es wurde Ende 2025 aufgrund mangelnder Finanzierung geschlossen.

Ein dezidiertes *Forschungsförderprogramm* für Teilhabeforschung existiert in Deutschland nicht, sie ist daher auf die allgemeine Forschungsförderung, u. a. von der Deutschen Forschungsgemeinschaft (DFG), angewiesen. Deren fachsystematische Förderlogik berücksichtigt jedoch nur bedingt die interdisziplinäre Ausrichtung der Teilhabeforschung.

### Soziale Dimension

Teilhabeforschung organisiert sich wesentlich über Netzwerke von Akteur:innen, die über die Grenzen einzelner Einrichtungen des Wissenschaftsbetriebs hinausgehen. Als ein zentrales Netzwerk wurde 2015 das bundesweite *Aktionsbündnis Teilhabeforschung *(im Folgenden: ABT) gegründet [5, 17]. Es handelt sich um einen Zusammenschluss von Wissenschaftler:innen, Menschen mit Behinderungen und ihren Interessenvertretungen, Fachgesellschaften sowie Fach- und Wohlfahrtsverbänden, der zu einer stärkeren Vernetzung und Förderung von Teilhabeforschung beitragen will. Inzwischen wurde das Netzwerk in das Format eines eingetragenen Vereins überführt, der wesentlich über Mitgliedsbeiträge finanziert wird. Eine institutionelle Förderung des ABT konnte bislang nicht erreicht werden.

Auf Initiative des ABT fand 2019 der erste *Kongress der Teilhabeforschung* statt. Seitdem dient er als interdisziplinäre bundesweite Plattform und wird in Kooperation mit wechselnden Hochschulen in etwa 2‑jährigem Rhythmus durchgeführt wird (2021, 2023, 2026). Bislang verzeichneten die Kongresse jeweils etwa 250 bis 300 Teilnehmende.

Das ABT weist somit in Teilen den Charakter einer Fachvereinigung auf. Unklar bleibt jedoch, inwiefern Teilhabeforschung für die wissenschaftlichen Mitglieder des ABT identitätsstiftend ist, da diese in der Regel zugleich anderen Fachgesellschaften angehören (z. B. Deutsche Gesellschaft für Rehabilitationswissenschaften – DGRW) oder sich in weiteren Scientific Communities bewegen (z. B. Disability Studies). Diese Durchlässigkeit der Grenzen zwischen Fachgesellschaften fördert zwar Offenheit in der Teilhabeforschung, steht jedoch ihrer klaren Profilierung und einer weiteren Institutionalisierung entgegen. Die Heterogenität der Mitgliedschaft, insbesondere der Einbezug von Expert:innen in eigener Sache, unterstützt die profilbildende Transdisziplinarität. Zugleich kann sie den Eindruck erwecken, dass es sich nicht um einen fachlich-wissenschaftlichen Zusammenschluss handelt – mit möglichen Nachteilen für die Anerkennung im Wissenschaftssystem.

Zur sozialen Dimension der Institutionalisierung gehören auch *wissenschaftliche Veröffentlichungen* und entsprechende *Publikationsorgane*. Im Unterschied z. B. zu den Disability Studies gibt es keine eigene Zeitschrift der Teilhabeforschung als zentralen Publikationsort mit wissenschaftlicher Reputation und Peer Review [18]. Publikationen, die der Teilhabeforschung zugerechnet werden können, finden sich verstreut in den Fachzeitschriften der an ihr beteiligten Disziplinen, ohne jedoch dezidiert als Beitrag der Teilhabeforschung erkennbar und sichtbar zu sein (einzelne Themenhefte stellen die Ausnahme dar [19–21]^2^, daneben die Zeitschrift *Teilhabe *[22]). Aus dem ersten *Kongress der Teilhabeforschung* ist der umfangreiche Sammelband *Teilhabeforschung – Konturen eines neuen Forschungsfeldes* hervorgegangen [23]. Er ist Teil der Schriftenreihe *Beiträge zur Teilhabeforschung*, in der bislang 6 Bände erschienen sind [24]. Die entstandene Publikationslandschaft ist Ausdruck einer zunehmenden wissenschaftlichen Sichtbarkeit, sie weist jedoch noch nicht die Selektivität eines eigenständigen Feldes auf. Das erschwert die Auffindbarkeit und damit auch die Profilbildung der *Teilhabeforschung* in der wissenschaftlichen Kommunikation.

### Epistemische Dimension

Ein zentraler Indikator für Fortschritte der Institutionalisierung von Forschungsfeldern ist die Herausbildung stabiler Wissensordnungen. Eine wichtige Rolle spielt dabei ein *gemeinsam geteiltes Begriffsverständnis*, das wissenschaftliche Kommunikation ermöglicht, Identität stiftet, Forschung vergleichbar und anschlussfähig macht und das Feld zugleich von benachbarten Feldern abgrenzt. Auch wenn sich ein gewisser Begriffskanon der Teilhabeforschung abzeichnet (z. B. Teilhabe, Behinderung, Partizipation, Inklusion, Exklusion, Selbstbestimmung, Barrieren), bleiben viele Begriffe mehrdeutig. Dies gilt auch für den Begriff der Teilhabe selbst, der den zentralen Bezugspunkt der Teilhabeforschung darstellt. Politisch-rechtlich beschreibt Teilhabe ein normatives Ziel; in der Praxis dient Teilhabe als professionelle, handlungsleitende Kategorie und für Betroffene ist sie Erfahrungs- und Ermächtigungsbegriff. Wissenschaftlich wird Teilhabe als analytischer Begriff in unterschiedlichen Theorietraditionen verortet, z. B. Capability Approach, Lebenslagen-Ansatz, Ökologische Entwicklungstheorie, Gerechtigkeitstheorien [23, S. 105–198]. Gerade die begriffliche Offenheit des Teilhabebegriffs scheint seine Anschlussfähigkeit für unterschiedliche wissenschaftliche Disziplinen zu fördern [25, S. 471]. Gleichwohl zeichnet sich ein gewisser Grundkonsens zu Kernelementen des Teilhabebegriffes ab, etwa zu seinen normativen Implikationen, seiner Mehrdimensionalität sowie seinem Subjekt- und Kontextbezug [26, 27].

Welche Fragen relevant sind, was als Wissen gilt und wie dieses Wissen erzeugt und bewertet wird, leitet die Teilhabeforschung aus konkreten Teilhabeproblemen von Menschen mit Behinderungen in ihrer Lebenswirklichkeit ab. Eine qualitativ codierte Auswertung der Programme der drei Kongresse der Teilhabeforschung^3^ zeigt übergreifend folgende thematischen Felder auf: Arbeit und Beschäftigung, Alltag, Wohnen und Sozialraum, Bildung, Gesundheit und Pflege, Politik und Recht, Familie und soziale Beziehungen, Alter/Lebensverlauf, Mobilität, Freizeit und Kultur.^4^ Methodologisch begegnet die auf den Kongressen repräsentierte Teilhabeforschung ihrem komplexen und normativ orientierten Gegenstandsbereich interdisziplinär und transdisziplinär. Programmhefte und Referent:innenlisten der Teilhabeforschungskongresse weisen die Beteiligung folgender Disziplinen aus: (Heil‑, Rehabilitations‑, Sonder‑)Pädagogik; Soziale Arbeit/Sozialpädagogik; Soziologie; Gesundheitswissenschaften/Public Health, teilweise mit (rehabilitations‑)medizinischem Bezug; Rechtswissenschaft, Politik‑/Verwaltungswissenschaft; Psychologie; Informatik/Technikgestaltung; Sportwissenschaft, Kulturwissenschaft; Philosophie.

*Transdisziplinarität* beschreibt eine Form der Wissensproduktion, die nicht nur disziplinäre Grenzen überschreitet, sondern die institutionellen und epistemischen Grenzen des Wissenschaftssystems selbst, indem sie mit nichtakademischen Wissensproduzent:innen zusammenwirkt [28, S. 16 f., 29]. Die Mitwirkung von Politik und Zivilgesellschaft, insbesondere von Menschen mit Behinderungen und ihren Interessenvertretungen, hat sich als zentral für die epistemische Kultur der Teilhabeforschung entwickelt [30, S. 4]. Mit der Beteiligung von Menschen mit Behinderungen als Wissenssubjekten an der Produktion und Bewertung von Wissen über Teilhabe begegnet die Teilhabeforschung dem tradierten epistemischen Ausschluss ihrer Erfahrungen und Deutungen als vermeintlich nicht relevant oder nicht glaubwürdig [31]. Bei einem breiten Spektrum empirischer *Methoden* [32] gewinnen insbesondere *partizipative Forschungsdesigns* an Bedeutung [33, 34]. Dies lässt sich exemplarisch an den bisherigen drei Kongressen der Teilhabeforschung zeigen: Partizipative Forschung ist jeweils Thema eines Hauptvortrags und einer Vielzahl an Einzelvorträgen und Forschungswerkstätten. Spätestens beim dritten Kongress zeigt sich partizipative Forschung als ein zentrales, querliegendes Anliegen.

### Dimension politisch-gesellschaftlicher Problemrelevanz

Die gesellschaftliche Bedeutung der Teilhabeforschung zeigt sich in ihrer politischen Relevanzsetzung. Ihre große Nähe zur Sozialpolitik erklärt zugleich ihre starke nationale Prägung. Vergleichbare Forschungsfelder gibt es im internationalen Raum nicht. Bereits an einer der frühen Vorläuferinitiativen zur Gründung des ABT lässt sich die politisch aufgeladene Entstehungsgeschichte von Teilhabeforschung festmachen [5, S. 4 f.]: Im Jahr 2011 richteten die fünf Fachverbände der Behindertenhilfe eine Fachtagung unter dem Titel „Teilhabeforschung jetzt! Eine Einladung an Forschung und Lehre“ aus. Das Interesse der Fachpraxis an Teilhabeforschung wurde hier unmittelbar mit der sozialpolitischen Ausgestaltung und Reform der Unterstützungssysteme für Menschen mit Behinderungen in Verbindung gebracht [35].

Nach der Gründung des ABT im Jahr 2015 gewann die Teilhabeforschung deutlich an Sichtbarkeit. Im „Nationalen Aktionsplan 2.0 der Bundesregierung zur UN-Behindertenrechtskonvention“ von 2016 findet sie als ein Instrumentalziel prominente Erwähnung [36]. Inzwischen widmet sich im BMAS ein eigenes Referat (Va1) der Teilhabeforschung, das auch die „Repräsentativbefragung zur Teilhabe von Menschen mit Behinderungen“ [37, 38] beauftragt und koordiniert. Dieser sog. Teilhabesurvey gilt als ein Meilenstein der Teilhabeforschung [39] und stellt eine wichtige Datenquelle für den Teilhabebericht der Bundesregierung dar. Mit Teilhabebericht und Teilhabesurvey verspricht sich die Bundesregierung, ihre durch die UN-BRK formulierten Verpflichtungen zur Sammlung von Forschungsdaten zur Umsetzung der Konvention (Art. 31 UN-BRK) zu erfüllen. Teilhabeforschung lässt sich damit auch als eine Antwort auf einen veränderten sozialpolitischen Bedarf interpretieren, der mit dem Anspruch einer menschenrechts- und evidenzbasierten Politik einhergeht [40].

Die hohe politische Relevanz der Teilhabeforschung drückt sich schließlich auch in der Ressourcenzuweisung aus: Für die politiknahe Teilhabeforschung wurden in den letzten Jahren Geldbeträge in zweistelliger Millionenhöhe bereitgestellt. Diese hohe Ressourcenzuweisung hatte allerdings nicht den Effekt einer Strukturverstärkung der Teilhabeforschung an Hochschulen. Die Ressortforschung wird vor allem von gewerblichen Forschungsinstituten getragen, vermutlich auch weil akademische Forschungsstellen nicht dieselbe Wettbewerbsfähigkeit aufweisen. Mit dieser Entwicklung ist die Gefahr verbunden, dass die politiknahe Forschung zu wenig mit den fachwissenschaftlichen Diskursen der Teilhabeforschung verschränkt ist [41].

### Zusammenfassung: Unterschiedliche Niveaus der Institutionalisierung

**Abb. 1:**
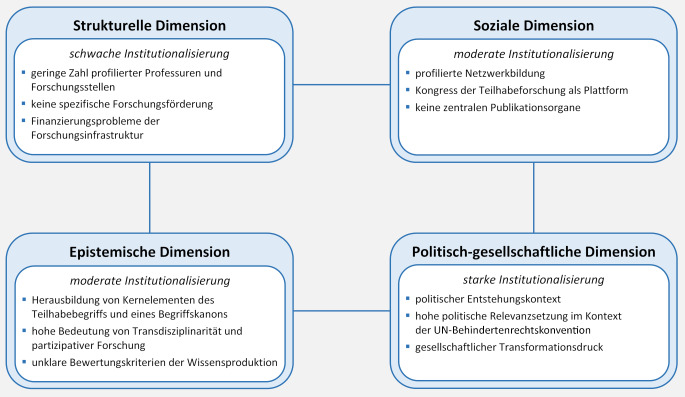
Institutionalisierung der Teilhabeforschung

Die Analyse der Entwicklung der Teilhabeforschung deutet auf unterschiedliche Niveaus der Institutionalisierung in den 4 beleuchteten Dimensionen hin (Abb. 1).

*Strukturell* zeigt sich, gemessen an der Zahl thematisch klar profilierter Professuren, Forschungsstellen und Programme der Forschungsförderung, das Bild einer nur schwach ausgeprägten Verankerung im deutschen Wissenschaftsbetrieb. Finanzierungsprobleme der Forschungsinfrastruktur weisen darauf hin, dass sich das Feld in einer instabilen Phase der Institutionalisierung befindet. Insgesamt bleibt die strukturelle Verortung der Teilhabeforschung, insbesondere im Verhältnis zu vergleichbaren interdisziplinären Forschungsfeldern wie den Gender Studies oder der Migrationsforschung, fragmentarisch.

Mit Blick auf die *soziale Dimension* zeigt sich eine stärker ausgeprägte Institutionalisierung im Sinne feldspezifischer Kommunikation. Dies kommt insbesondere in der profilierten Netzwerkbildung mit einer charakteristischen Akteurskonstellation aus Wissenschaft, Politik, Fachpraxis und Interessenvertretungen zum Ausdruck. Auch die Etablierung eines eigenen Kongresses ist Indikator einer fortgeschrittenen Institutionalisierung in sozialer Hinsicht. Andererseits fehlen – trotz der zunehmenden Zahl an Buchpublikationen und thematisch fokussierten Sonderheften – eigenständige Fachzeitschriften, die zur wissenschaftlichen Sichtbarkeit und Auffindbarkeit beitragen und zugleich eine zentrale Rolle für Qualitätssicherung sowie Publikationsrenommee spielen.

Für die *epistemischen Grundlagen* von Teilhabeforschung lässt sich eine moderate Konsolidierung feststellen. Als offener Diskursraum vermag das Forschungsfeld heterogene begriffliche und theoretische Perspektiven zu integrieren, ohne sie vollständig zu vereinheitlichen. Zugleich bilden sich neben klaren thematischen Feldern ein gewisser Begriffskanon sowie eine Verständigung auf zentrale Elemente im Begriffsverständnis von Teilhabe heraus. Transdisziplinarität als Co-Produktion von Wissen mit nichtakademischen Akteur:innen, partizipative Forschungsansätze und die explizite Auseinandersetzung mit den Bedingungen ihrer Wissensproduktion zeigen sich als zentrale Merkmale der epistemischen Kultur von Teilhabeforschung. Diese Offenheit und Innovationskraft bringen jedoch auch Unsicherheiten für die Institutionalisierung mit sich, etwa im Hinblick auf wissenschaftliche Bewertungssysteme und Reputation [42].

Die Teilhabeforschung profitiert stark vom politischen Kontext bzw. von der hohen *gesellschaftlichen und politischen Bedeutung*, die ihr angesichts der UN-BRK und des gesellschaftlichen Transformationsdrucks unter den Vorzeichen eines wachsenden Bevölkerungsanteils mit Behinderungen zukommt. Die Befunde zeugen von einem hohen Institutionalisierungsgrad, sind jedoch auch ambivalent zu betrachten: Die externe Relevanz und der hohe Anwendungsbezug erzeugen eine hohe Nachfrage nach Teilhabeforschung und erleichtern die Ressourcenmobilisierung. Sie wirken sich jedoch bislang nur in begrenztem Maße strukturverstärkend aus. Zudem ist eine große Nähe der Wissenschaft zur (Regierungs‑)Politik zugleich Ausdruck von Anerkennung und eine mögliche Gefahr für die Unabhängigkeit und kritische Distanz von Forschung [25, S. 471]. Gleiches gilt für eine allzu große Nähe zu Interessenvertretungen und politischen Bewegungen behinderter Menschen, denn parteiische Forschung kann mit den Standards guten wissenschaftlichen Arbeitens in Konflikt geraten [43]. Schließlich gefährdet der gegenwärtige Rechtsruck in Politik und Gesellschaft [44], einschließlich behindertenfeindlicher Positionen [45], die politische Legitimation und Förderung der Teilhabeforschung.

## Fazit

Teilhabeforschung befindet sich in einer dynamischen Phase der Feldbildung und -verbreitung. In den vergangenen Jahren sind deutliche Fortschritte ihrer Institutionalisierung zu beobachten, insbesondere mit Blick auf ihre Netzwerkbildung, die thematischen Verdichtungen und methodologischen Standards sowie ihre politisch-gesellschaftliche Problemrelevanz. Zugleich ist das Feld jedoch noch nicht vollständig stabilisiert. Die weitere Konsolidierung der Teilhabeforschung hängt maßgeblich davon ab, ob dauerhafte strukturelle Verankerungen und tragfähige Bewertungsstandards entwickelt werden.

## Data Availability

Alle dieser Arbeit zugrunde liegenden Daten sind in diesem Artikel enthalten.

## Footnotes

1 Relevant ist auch die strukturelle Verankerung von Teilhabeforschung als Lehr- und Qualifizierungsgebiet (z. B. Studiengänge, Graduiertenkollegs), die jedoch im Rahmen dieses Beitrages nicht beleuchtet wird.

2 Für eine genaue Untersuchung der in den Veröffentlichungen der Teilhabeforschung behandelten Themen wäre eine bibliometrische Analyse notwendig.

3 Die Auswertung erfolgte KI-unterstützt mit ChatGPT (5.2).

4 Dabei überwiegen bei allen Kongressen empirische (Projekt‑)Beiträge (60–70 %), gefolgt von methodischen Beiträgen (20–25 %) und theoretisch-konzeptionellen Beiträgen (10–15 %).
